# Physician associate preceptorship: Experience of a novel programme in Inverness

**DOI:** 10.1016/j.fhj.2024.100200

**Published:** 2024-10-24

**Authors:** Liam Allan, Duncan Scott, Ed Paterson, Tony Golabek

**Affiliations:** a Ward 7B, Raigmore Hospital, Inverness, IV23UJ; b Ward GA, Raigmore Hospital, Inverness, IV23UJ; c Management Corridor, Raigmore Hospital, Inverness, IV23UJ

**Keywords:** Physician associate, Internship, Preceptorship

## Abstract

Physician associates (PAs) are a developing profession in the UK. The Faculty of Physician Associates (FPA) recommends the establishment of local preceptorship programmes for PAs in the first year of practice who have successfully passed the PA national exam. Limited evidence currently exists around the evaluation of such local programmes and the experience of the intern PAs involved.

Over the course of a 12-month period, four intern PAs completed a structured preceptorship comprising clinical supervision, teaching and mentoring. Upon completion of this programme, qualitative data were recorded and analysed to influence the development of the preceptorship in future.

In conclusion, a structured preceptorship has been shown to be a useful method of improving clinician confidence and competence while offering a supported working environment for newly qualified PAs.

## Highlights

• Physician Associates in the UK could benefit from a structured programme of learning post qualification, however this is not currently mandated.
• We share our experience of a structured preceptorship model for Physician Associates in our area, which brought improved practitioner confidence in addition to providing assurance of quality.
• We advocate for a national level framework to support standardisation of preceptorship for all Physician Associates working in the UK.

## Introduction

Physician associates (PAs) are a developing professional group in the UK; however, evidence pertaining to successful preceptorship within healthcare teams is limited.^1^ Evidence suggests that successful integration of first-year qualified PAs requires role clarity, skill development, role modelling and championing by experienced healthcare professionals.^1^ Limited data highlight that delivering a structured preceptorship contributes to positive working relations, promotes safe working practices and fosters professional growth.^2^ The Faculty of Physician Associates (FPA) currently advocates for the completion of a preceptorship in the first year of employment, which should encompass close supervision, experiential learning and maintenance of a portfolio of evidence.^3^ Significant debate has taken place with regards to the integration of PAs in the UK, including their role, regulation and professional identity.^4^ National-level frameworks, robust local governance and supported role development are vital in ensuring safe working practices and development of this workforce.^5^ Newly qualified PAs in Raigmore Hospital undergo a locally developed internship, ensuring robust supervision with ongoing assessment of clinical competence while promoting a culture of professional support.

Our aim was to implement a structured framework to allow intern PAs (iPAs) to demonstrate learning and gain confidence in the clinical environment over a period of 12 months. During this period, four iPAs were employed with a background level of experience ranging from employment in a healthcare support role, alongside study, to experience as a PA in a different healthcare system. All iPAs were suitably qualified, having completed an honours level bachelor's degree in a healthcare-related subject followed by a master's degree in PA studies. Finally, all iPAs had successfully passed the PA national examination (PANE) and were registered on the PA Managed Voluntary Register (PAMVR).

## Method

A structured preceptorship programme was developed for iPAs starting work in the medical division. iPAs were employed in general medicine for 12 months in a non-rotational post, clinically supervised by a named consultant supervisor in addition to dedicated programme supervision. iPAs operated at the level of a PA as part of the multiprofessional care team. Core tasks included supporting clinical care on inpatient wards, adding flexible capacity to existing clinical teams and developing responsibility for a specific area of patient care. It was felt that continuity was a key benefit that this role could bring to multiprofessional teams, and so iPAs were not rotated during their preceptorship to allow for continuity of care and the fostering of collaborative interprofessional relationships in the clinical environment.

Curricular outcomes were matched to the recommendations of the General Medical Council (GMC) and FPA and offered to iPAs employed following successful completion of the PANE.^6^, ^7^, ^8^ Enrolled iPAs were given a booklet of GMC PA learning outcomes^6^, grouped by key domains, to be demonstrated over the course of the internship and confidential access to a locally developed personal online log. Assessed domains included professional behaviour and trust, safety and quality, clinical care and professional capabilities. The online log contained access to a clinical skills log, space to undertake reflective writing and a central point of communication with programme supervisors. Workplace-based assessments (WBA) were recorded on NHS Scotland's TURAS ePortfolio^9^ and made available to appraisers. Objective attainment was assessed using a mixed method of clinical skills log book, WBA, multisource feedback (MSF) and four pieces of reflective writing.

Competence in clinical skills was assessed using a clinical skills log book, encompassing 22 core PA clinical skills identified by the FPA.^10^ In order to demonstrate competence, a directly observed procedural skill (DOPS) record was completed by an appropriately qualified member of staff for each skill. Furthermore, iPAs were required to undergo eight mini-clinical evaluation exercises (mini-CEX) and eight case-based discussions (CBD) to be carried out by a range of experienced members of the multiprofessional care team, predominantly senior medical staff. In order to assess behaviours in the clinical environment, two team assessments were obtained at 6 months and 12 months of the preceptorship. Each assessment required a minimum of six documented MSF from a range of members of the clinical team with no more than two from a single professional group. Finally, iPAs were required to complete four pieces of reflective writing relating to the four assessed domains over the course of the preceptorship.

Assessment of iPAs was undertaken by means of regular supervision meetings with the programme supervision team, separate from the iPA consultant clinical supervisor. This team comprised a senior physician, experienced PA – defined as having 5 years post-qualification experience, holding a postgraduate qualification in clinical education and demonstrable experience in the delivery of educational programmes – and a service manager at 3- and 6-month intervals, with final viva voce assessment on completion of the programme.^11^ The aim of the viva voce was to ensure that a panel of members of the supervision team and additional external experienced PA were satisfied that iPAs demonstrated competence in operating safely at the level of a PA. This assessment method encouraged reflection in iPAs, demonstrating professional development over the course of the preceptorship and a commitment to lifelong learning. In order to objectively assess this, a question list was agreed and marking rubric developed, which was completed by the assessment team with an opportunity for feedback to iPAs following evaluation. Throughout the course of the preceptorship, iPAs underwent a mandatory full day induction including clinical topics and corporate induction, regular team teaching and engagement with quality improvement activities. All iPAs completed the Resuscitation Council Advanced Life Support course during this preceptorship to ensure competence in the safe management of clinical emergencies.

Following completion of the programme, qualitative data were collected by means of a feedback questionnaire completed by enrolled iPAs, with a key overview metric being iPA confidence in the clinical environment. Data were then coded into key themes and analysed, with feedback used to review and streamline the overall process.

## Results

iPAs reported an average 23% improvement in confidence working in the clinical environment over the period of the preceptorship. This was assessed using a modified Likert scale.^12^ Notably, iPA self-assessment of confidence prior to undertaking the preceptorship was scored at 3.25 out of 5, increasing to 4.0 out of 5 following completion. The Constant Comparative Method^13^ was used to analyse qualitative data (Table 1) with overarching themes identified relating to four domains: clinical care, multidisciplinary team (MDT) integration, support and educational opportunities.

**Table 1:** Thematic analysis of qualitative data.

### Clinical care

iPAs reported satisfaction in delivering quality patient care, especially for those with complex clinical and discharge requirements:

‘*Getting patients safely discharged home especially after long rehab periods…”*

Additionally, being a continuous presence in a clinical area was found to be satisfying:

‘*Bonding with patients and being a constant for them…”*

### MDT integration

iPAs reported a feeling of supported inclusive working within the MDT, however there were occasions where the role was not well understood by service users and staff:

‘*Working with a great team … the wider MDT, has been rewarding*’

‘*Having to explain the PA role to patients, their families and staff … gaining their understanding and trust*’

Some found that working with the wider MDT supported a culture of patient safety and quality improvement:

‘*… through building rapport with your seniors … you can increase the safety of your patients*’

### Support

All iPAs reported a feeling of appropriate and accessible support in the clinical environment:

‘*I found [members of the clinical team] particularly supportive and in providing opportunity to complete the folio …*’

Additionally, most found programme supervision especially helpful when dealing with professional tension:

‘*Support in the face of negative attitude towards the PA*’

### Educational opportunities

Although some would prefer more profession-specific teaching, most found existing teaching to be appropriate and useful, especially when delivered by role models:

‘*[Experienced PA] has also provided some great clinical and soft-skills teaching*’

All iPAs reported appropriate and helpful access to workplace-based teaching:

‘*… some excellent junior doctors … taught me a lot*’

### Assessor confidence

An important element of iPA final competence assessment was that the panel were satisfied that iPAs could operate safely at the level of PA in the clinical environment given evidence provided throughout the preceptorship. To ensure a robust process, the assessment panel were surveyed with the question ‘how confident are you that the intern programme delivered competent clinicians that will deliver in the clinical environment?’ and anonymous responses collated. A modified Likert scale was used to grade responses from members of the assessment panel (n=3). 100% of respondents indicated that they were ‘very confident’ that the intern programme delivered competent clinicians that will deliver in the clinical environment.

## Discussion

The establishment of a structured preceptorship for iPAs has been a valuable quality assurance intervention and has significantly improved the clinical confidence of first year qualified PAs in our area. Additionally, our data imply that the experience has been positive overall for iPAs, especially through delivering quality patient care in conjunction with the wider multidisciplinary team. Educational opportunities and support from supervisors, especially during a time of professional challenge, have been important to iPAs. However, some key learning has been highlighted, which serves to improve and streamline the preceptorship going forward.

It is recognised that it would be useful to undertake patient experience surveys to gain a clear understanding of how patients perceive their care from this group of healthcare professionals, including understanding of role and identity. This is something that will be incorporated as this programme develops.

Additionally, this process has been useful in formalising governance and supervision arrangements for iPAs in our area and has supported individual development, ultimately improving iPA confidence in the clinical environment. The structure serves to foster a supportive culture for iPAs and a means of assuring the quality of early career PA practice within clinical teams.

## Conclusion

While there is not a universally agreed approach to preceptorship, it is imperative that healthcare leaders promote safety and quality in post-qualification PA practice. Structured preceptorship brings the opportunity to improve iPA confidence in clinical practice and quality assurance of the clinical capabilities of this professional group. Our study has highlighted a potential method of achieving this, which is robust and adaptable to various clinical environments.

## Ethics approval and consent to participate

Ethics approval was not required for this piece describing a curriculum for preceptor physician associates with feedback. All participation was voluntary and written consent was not required due to minimal risks.

## Funding

No funding or research grants were received in the course of study, research or assembly of the manuscript.

## Data availability

Raw data are available on request for this paper.

## CRediT authorship contribution statement

**Liam Allan:** Writing – review & editing, Writing – original draft, Validation, Resources, Project administration, Methodology, Investigation, Formal analysis, Data curation, Conceptualization. **Duncan Scott:** Writing – review & editing, Validation, Supervision, Formal analysis, Conceptualization. **Ed Paterson:** Writing – review & editing. **Tony Golabek:** Project administration.

## Declaration of competing interest

Liam Allan is a Physician Associate in Respiratory Medicine and Medical Associate Professions Teaching Co-ordinator at NHS Highland. If there are other authors, they declare that they have no known competing financial interests or personal relationships that could have appeared to influence the work reported in this paper.

## Appendix B Supplementary materials

Image, application 1

Image, application 2

Image, application 3
