# Supplementary material for: Physician associate preceptorship: Experience of a novel programme in Inverness
Source: Future Healthc J. 2024 Oct 24;11(4):100200. doi: 10.1016/j.fhj.2024.100200 (PMC11600754; doi:10.1016/j.fhj.2024.100200)
Supplement: Supplementary file 1 [file mmc1.pdf]

Physician Associate Intern

Core Competencies Log

**Name:**
**Registration number:**

V1DEC22

Signatories

It is the responsibility of the Physician Associate to ensure that those signing off on individual competencies are themselves competent and appropriately experienced to sign off on each item. This may be subject to verification by means of quality assurance. All competencies signed must be supported by a DOPS form on TURAS ePortfolio.

Signatory Details

Name	Designation	Registration number	Signature

This booklet is to be used in conjunction with '*Curriculum for the Physician Associate intern year*'

Matrix and timeline for competency attainment

	**Component**	**Supervisor meeting 1** (3 months)	**Supervisor meeting 2** (6 months)	**Supervisor meeting 3** (1 year)
**Clinical Skills**	Baseline observations			
	Surgical scrubbing-up			
	CPR training at ILS level			
	CPR training at ALS			
	Venepuncture			
	IV Cannulation			
	ABG sampling and interpretation			
	Blood culture acquisition			
	Capillary blood glucose			
	Peak flow testing			
	Urinalysis			
	12 lead ECG			
	Viral throat swab			
	Male urinary catheterisation			
	Female urinary catheterisation			
	Chest X-ray interpretation			
	Echocardiogram result interpretation			
	Blood testing interpretation			
	NGT insertion			
	Oxygen administration			
	Inhaled therapy administration			
	drug dose calculation			
	Set up an infusion			
**Medicines administration**	Parenteral			
	Subcutaneous			
	Intramuscular			
	Intravenous			
**Local anaesthetic administration**	Topical			
	Intradermal			
	Urethral			
**Portfolio**	1 Mini-CEX and 1 CBD			
	1 mini-CEX and 1 CBD			
	2 mini-CEX and 2 CBD			
	Multi Source Feedback			
	Multi Source Feedback			
	Reflection 1: Professional behaviour and trust			
	Reflection 2: Professional capabilities			
	Reflection 3: Clinical Care			
	Reflection 4: Safety and Quality			

All observable components in clinical skills, medicines administration and local anaesthetic administration require to also have a DOPS form completed for each.

Core Clinical Practice

Component	Date competent	Evidence	Signature
Baseline observations (to include temperature, respiratory rate, heart rate, blood pressure, oxygen saturations, urine output) with appropriate recording			
Perform surgical scrubbing and gowning			
Participate or train in CPR to the level of Immediate life support (ILS)			
Participate or train in CPR to the level of Advanced Life Support (ALS)			

Core Clinical Practical Procedures

Component	Date competent	Evidence	Signature
Carry out venepuncture			
Carry out intravenous cannulation			
Carry out arterial blood gas sampling and acid base sampling from the radial artery in adults			
Accurately interpret arterial blood gas result			
Take blood culture of infectious organisms			
Measure capillary blood glucose			
Explain to a patient how to perform a peak expiratory flow test, assess that it is performed adequately and interpret results			
Carry out a urine multi-dipstick test and be able to interpret results			
Carry out a 12-lead electrocardiogram and be able to interpret results			
Take and/or instruct patients how to take a viral throat swab			
Carry out male urinary catheterisation			
Carry out female urinary catheterisation			
Interpret a chest x-ray			
Interpret an echocardiogram result			
Interpret serological testing results			

Core Therapeutic Procedures

Component	Date competent	Evidence	Signature
Carry out nasogastric tube placement including confirmation of safe usage (e.g. on x-ray or aspirate).			
Recommend and administer oxygen appropriately			
Instruct patients in the use of devices for inhaled medications			
Undertake basic drug calculations			
Set up an infusion			
Prepare and administer parenteral medications			
Prepare and administer subcutaneous medications			
Prepare and administer intramuscular medications			
Prepare and administer intravenous medications			
Administer topic local anaesthetic			
Administer subcutaneous local anaesthetic			
Administer urethral local anaesthetic			
Carry out wound care and closure, including suturing with simple interrupted suture and dressing			

Reflecting on Practice

Reflection is a useful method of considering your practice and how you might approach situations in future. To be a reflective practitioner is to continue life long experiential learning and utilise opportunities for improving your own practice. You are asked to complete 4 reflections on each of the following topics: professional behaviour and trust, professional capabilities, clinical care, safety and quality.

You should choose a reflective model of your choosing to complete these and demonstrate you have met the outcomes referred to under each theme in the document *Competencies for the Physician Associate Intern Year.* These will be reviewed and appraised by means of viva voce assessment at the end of your intern year. Please carry these out using any format you wish and upload completed reflections to your TURAS eportfolio for review.

Reflection	Topic	Date completed
1	Professional behaviour and trust	
2	Professional capabilities	
3	Clinical care	
4	Safety and quality	

Practical Clinical Assessment

Ongoing clinical assessments from colleagues allow you to demonstrate your ability to provide high quality care to your patients. You will continue to evidence this throughout your practice as a PA.

Please record all Mini-CEX and DOPS on TURAS. You may also record completion of this below. We expect you to complete a **minimum** of 8 Mini-CEX and 8 CBD by the end of the intern year. Additionally, you should complete multi source feedback at 6 months and one year of service.
