# Supplementary material for: Physician associate preceptorship: Experience of a novel programme in Inverness
Source: Future Healthc J. 2024 Oct 24;11(4):100200. doi: 10.1016/j.fhj.2024.100200 (PMC11600754; doi:10.1016/j.fhj.2024.100200)
Supplement: Supplementary file 2 [file mmc2.docx]

Viva voce assessment questions

- How do you feel you have developed as a professional this year and what will you do to continue this development?
- Tell us about your experience of working in the multi-professional clinical team, including any challenges and opportunities this has involved?
- Tell us about a time that you delivered care that you are proud of?
- How do you ensure that you practice safely and that the care you deliver is of the required standard?
- What do you see as the next steps and how can we help?
