# Supplementary material for: Physician associate preceptorship: Experience of a novel programme in Inverness
Source: Future Healthc J. 2024 Oct 24;11(4):100200. doi: 10.1016/j.fhj.2024.100200 (PMC11600754; doi:10.1016/j.fhj.2024.100200)
Supplement: Supplementary file 3 [file mmc3.docx]

**Physician Associate preceptor programme final review**

The viva voce is in a conversational review which allows intern PAs to demonstrate that they meet the learning objectives set by the preceptor programme. Assessors will ask open ended questions which allow the intern to reflect on their practice. The aim of this assessment is to elicit holistic evidence of the intern’s learning experience, confirm attainment of the programme learning objectives and reflect on successes and difficulties encountered in undertaking the programme.

For a candidate to successfully complete this assessment, assessors must be content that the PA can demonstrate evidence of safe clinical practice, development as a professional and commitment to career long learning. The assessment will last 30 minutes and will take place in a confidential environment.

Assessment faculty will comprise of the PA intern supervision team in addition to an external team member (an experienced PA not directly involved in the intern programme). Faculty will decide on one of the following outcomes for candidates based on viva voce assessment based on the attached Rubric:

**Outcome 1** – Candidate meets criteria for unconditional completion of preceptor programme. Outcome 1 recorded in all domains.

**Outcome 2** – Candidate meets criteria for conditional completion of preceptor programme (N.B. conditions and follow up timescale must be clearly communicated) Outcome 2 recorded in some domains with some in outcome 1, no outcome 3 recorded.

**Outcome 3** – Candidate does not meet the criteria for successful completion of the preceptor programme. Outcome 3 recorded in any domain.

Where an outcome of 2 or 3 is recorded, a candidate will be offered an individualised supportive meeting whereby a clear strategy can be organised to support them to attain the learning outcomes and formally complete the internship.

| **Physician Associate Intern viva voce assessment rubric** | | **Outcome 1** | **Outcome 2** | **Outcome 3** |
| --- | --- | --- | --- | --- |
|  |  | *Candidate meets criteria for unconditional completion of the preceptor programme* | *Candidate meets criteria for conditional completion of the preceptor programme* | *Candidate does not meet the criteria for successful completion of preceptor programme* |
| **Domain** | **Key outcome theme** |  |  |  |
| **Professional behaviour and trust** | Professional duties and responsibilities | Competently reflects on key outcome themes, demonstrating attainment and engagement with appropriate local and national policy | Reflects on key outcome themes, but cannot demonstrate attainment or engagement with appropriate local and national policy | Unable to reflect on key outcome themes, demonstrate attainment of objective or awareness of local or national policy |
|  | Being honest and trust worthy |  |  |  |
|  | Partnership working and supported decision-making |  |  |  |
|  | Consent and mental capacity |  |  |  |
|  | Personal responsibilities and wellbeing |  |  |  |
|  | Legal responsibilities |  |  |  |
| **Professional capabilities** | Holistic and integrated care | Competently reflects on key outcome themes, demonstrating attainment and engagement with appropriate local and national policy | Reflects on key outcome themes, but cannot demonstrate attainment or engagement with appropriate local and national policy | Unable to reflect on key outcome themes, demonstrate attainment of objective or awareness of local or national policy |
|  | Health promotion and illness prevention |  |  |  |
|  | Communication |  |  |  |
|  | Safeguarding |  |  |  |
|  | Working in multi-professional teams |  |  |  |
|  | Respect for colleagues |  |  |  |
|  | Management and leadership |  |  |  |
|  | Manage time and workload |  |  |  |
|  | Lifelong learning |  |  |  |
| **Clinical care** | Diagnosis and effective consultations | Competently reflects on key outcome themes, demonstrating attainment and engagement with appropriate local and national policy | Reflects on key outcome themes, but cannot demonstrate attainment or engagement with appropriate local and national policy | Unable to reflect on key outcome themes, demonstrate attainment of objective or awareness of local or national policy |
|  | Medical management |  |  |  |
|  | Emergency and acute care |  |  |  |
|  | Complex care and uncertainty |  |  |  |
|  | Managing prescribed medicines safely |  |  |  |
|  | Using information effectively and safely |  |  |  |
| **Safety and quality** | Patient safety and quality improvement | Competently reflects on key outcome themes, demonstrating attainment and engagement with appropriate local and national policy | Reflects on key outcome themes, but cannot demonstrate attainment or engagement with appropriate local and national policy | Unable to reflect on key outcome themes, demonstrate attainment of objective or awareness of local or national policy |
|  | Clinical research and scholarship |  |  |  |
|  | Healthcare resource management |  |  |  |
|  | Teaching and learning |  |  |  |
| **Clinical skills portfolio** | | Completed skills portfolio with no significant concerns raised by clinical supervisor | Partially completed skills portfolio with no significant concerns raised by clinical supervisor | Grossly incomplete skills portfolio **and/or** significant concerns raised by clinical supervisor |
